# The collaborative effect of scientific meetings: A study of the International Milk Genomics Consortium

**DOI:** 10.1371/journal.pone.0201637

**Published:** 2018-08-22

**Authors:** Eric Kwok, Matthew Porter, Ian Korf, Gonca Pasin, J. Bruce German, Danielle G. Lemay

**Affiliations:** 1 Genome Center, University of California Davis, Davis, California, United States of America; 2 Department of Molecular and Cellular Biology, University of California Davis, Davis, California, United States of America; 3 California Dairy Research Foundation, Davis, California, United States of America; 4 Department of Food Science and Technology, University of California Davis, Davis, California, United States of America; 5 USDA-ARS Western Human Nutrition Research Center, Davis, California, United States of America; Institut Català de Paleoecologia Humana i Evolució Social (IPHES), SPAIN

## Abstract

Collaboration among scientists has a major influence on scientific progress. Such collaboration often results from scientific meetings, where scientists gather to present and discuss their research and to meet potential collaborators. However, most scientific meetings have inherent biases, such as the availability of research funding or the selection bias of professional societies that make it difficult to study the effect of the meeting per se on scientific productivity. To evaluate the effects of scientific meetings on collaboration and progress independent of these biases, we conducted a study of the annual symposia held by the International Milk Genomics Consortium (IMGC) over a 12-year period. In our study, we conducted permutation testing to analyze the effectiveness of the IMGC in facilitating collaboration and productivity in a community of milk scientists who were meeting attendees relative to non-attendees. Using the number of co-authorships on published papers as a measure of collaboration, our analysis revealed that scientists who attended the symposium were associated with more collaboration than were scientists who did not attend. Furthermore, we evaluated the scientific progress of consortium attendees by analyzing publication rate and article impact. We found that IMGC attendees, in addition to being more collaborative, were also more productive and influential than were non-attendees who published in the same field. The results of our study suggest that the annual symposium encouraged interactions among disparate scientists and increased research productivity, exemplifying the positive effect of scientific meetings on both collaboration and progress.

## Introduction

The complexity of scientific problems, especially in the life sciences, often requires multidisciplinary expertise within structures based on teams of researchers. Besides access to expertise, scientists also collaborate to improve access to funds and resources, to advance professionally, to improve efficiency and make more rapid progress, to enhance their ability to tackle bigger problems, and to boost productivity [1]. Evidence documents that research teams now produce more publications than solo authors and these team papers also have higher impact, suggesting that the pattern of knowledge creation has fundamentally changed in the latter part of the 20th century [2]. Furthermore, the emergence of such highly collaborative work has prompted researchers to conduct studies on the mechanisms of scientific collaboration [3]. These studies, including those that analyze records from as far back as 1900 [4], reveal that more collaboration often results in the publication of higher impact articles [5]. Such studies could eventually help inform organizational decisions that could facilitate collaboration across disciplines [6], institutions [7], or countries [8].

In certain research areas defined by modern enabling technologies, such as genome science and genetics, the traditional size of groups typically required to produce a single research article has evolved into much larger teams, or consortia. A simple search for the word “consortium” in the PubMed database [9], which is a database of biomedical literature, reveals tens of hits in the 1970s and 1980s, hundreds of annual hits in the 1990s and early 2000s, and thousands of hits beginning in 2008 with over 5000 hits in 2016. A review of the earliest hits of the word “consortium” in the PubMed database suggests that consortia at that time were mainly for the purposes of health education or delivery of health services. With the arrival of the International Human Genome Sequencing Consortium [10], which produced the first human genome, and the International HapMap Consortium [11], which published the major human haplotypes, and newly assembled genetic tools, these health delivery-driven consortia were naturally extended to include genetics studies. The sequencing and assembly of the first mammalian genomes required consortia because of the sheer workload. Similarly, the consortium-style science for genome-wide association studies (GWAS) is largely driven by the need to include more study subjects for greater power to detect significant genetic variants. Indeed, the need to include more and more study subjects is driving new models of collaboration, such as the UK10K Project Consortium, in which the genetic code of 10,000 people with pre-existing phenotype information is being finely mapped and linked to disease risk [12].

In contrast, consortium science is less common among agricultural scientists, with the exception of the genome projects of agriculturally important species. The word “consortium” in the CAB abstracts database [13], which is a database of agricultural literature, has ten times fewer hits in 2016 than the PubMed database of biomedical literature, even though some of these hits are overlaps with those indexed in PubMed. Additionally, many of the non-overlapping hits are for microbial consortia rather than consortia of scientists.

It was within this context of limited consortium science in agricultural spheres that the International Milk Genomics Consortium (IMGC) was founded in 2004. The IMGC is sponsored by industry members and dairy organizations around the world, and their collective funds are managed on behalf of the sponsors by the California Dairy Research Foundation. However, there have been no funds within the consortium apart from those supporting the symposia. The IMGC was founded to avoid the bias of research support in part because of the potential influence of industrial funding on the scientific agenda. The stated mission of the IMGC is “to provide a collaborative, interactive and pre-competitive platform for scientific community and industry to accelerate the understanding of the biological processes underlying mammalian milk genomics and facilitate the transition of that knowledge into usable commercial benefits for industry.” In short, the IMGC was assembled to facilitate collaboration among milk scientists. Besides providing a symposium for scientists working on bovine milk genomics and genetics, the IMGC also intended to foster collaborations among scientists working in different fields, and between academia and industry.

Evaluation of the relative success of cross-disciplinary collaborations among scientists is of scholarly interest and yet it is virtually impossible to isolate collaboration from the evident bias of funding due to the nature of societal organizations that explicitly fund research within their members. Relatively few consortia exist that are independent of that bias. Consortia for the sequencing and analysis of animal or plant genomes are explicitly funded for that scientific project. For example, the International Lettuce Genomics Consortium is funded to sequence, assemble, and annotate the reference genomes of two wild lettuce species. In contrast, the IMGC has not funded scientific research. This makes the IMGC a unique case study to investigate the advantages of scientific meetings independent of scientific funding.

Many factors have been studied to determine whether they influence scientific productivity. These factors include demographics (age, gender, race), family-related factors (marital status, children), human capital (PhD program, dissertation subfield), opportunity costs (teaching and committee service), working environment, and professional variables (e.g. frequency of conference presentation) (reviewed in Hesli and Lee [14], Table 1). Hesli and Lee evaluated the effects of these variables on the number of published articles using a multivariate analysis of results from survey respondents of the American Political Science Association in 2009 [14]. Frequency of conference attendance was a significant positive predictor of publication output, but not as strong of a predictor as other factors such as gender, faculty rank, and PhD program. Other studies point to an effect of conference attendance on publication output. In an analysis of faculty publication patterns in ten different countries, membership in professional associations or attendance at their annual meetings was an important predictor of article productivity in all ten academic systems [15]. In a study of approximately half of the population of young scientists in Croatia, the most significant predictor of the total number of publications was most strongly determined by one factor: attendance at international scientific conferences abroad [16]. Barnes and Beaulieu [17] evaluated the effect of a National Science Foundation-funded annual conference in political methodology for women on the productivity and found that women who attended the conference had higher average journal article submissions per year than women who did not. Kyvik and Larsen [18] studied the effect of conference attendance on research performance of researchers from small countries: in all fields of learning, they found that those who were invited to present a paper by conference organizers were the most productive, followed by those who presented a paper without invitation followed by those who attended without presenting.

**Table 1:** Mean, median, and standard deviation for number of collaborators, rates of publication, and article efficiencies before and after first IMGC attendance (*n* = 311).

Metric	Mean	Median	Standard Deviation
Number of Collaborators Before First IMGC Attendance	0.50	0	1.55
Number of Collaborators After First IMGC Attendance	5.27	2	8.24
Rate of Publication Before First IMGC Attendance (Papers Per Year)	0.25	0	0.46
Rate of Publication After First IMGC Attendance (Papers Per Year)	1.10	0.75	1.18
Article Efficiency Before First IMGC Attendance	1.12	0	2.37
Article Efficiency After First IMGC Attendance	3.69	2.5	4.98

In many studies of the effect of conferences on researcher productivity, there is a natural selection bias in favor of high-producing researchers. Compared with other scientific meetings, there are unusual characteristics of the annual IMGC symposium that make it a unique case study to understand the impact of scientific meetings on the careers of individual scientists. Unlike professional societies, there is no membership application for the IMGC that would exclude attendees who are not yet established in the field. There are no professional requirements to attend the conference or to submit an abstract. The requirements for poster presentation are minimal; they are reviewed for scientific quality, but rejection of poster abstracts is extremely rare. There is no requirement that the attendees have any track record at all in the field, nor that they even be scientists. Indeed, some attendees are dairy farmers or non-publishing industry representatives. There are also no dues, which can be a financial barrier. At the annual meeting, there is no requirement that the work to be presented has been already been published, nor is there a requirement that it is not yet published. There is no funding for any particular research project to be conducted by the IMGC. The annual symposium is a small meeting (approx. 70–120 attendees) designed to increase networking opportunities and to bring in new attendees (approx. 40 each year). It is also an international conference with strong attendance from Europe, North America, and Australia/New Zealand, and alternating locations on a different continent each year. It is truly designed as a networking event, but without the gatekeeping of professional societies. Together, these characteristics reduce the selection bias that would be true of scientific meetings that are part of professional societies or funded projects, and it provides a unique case study to evaluate the impact of meetings on both the group as a whole and on the careers of individual scientists.

In the current study, we sought to evaluate whether the IMGC—a consortium not tied to research funds or to a professional scientific society—was successful at facilitating collaboration. To determine the progress of the IMGC towards scientific collaboration, we evaluated collaboration metrics derived from publication records, such as the number of co-authorships and the rate of publication, among attendees of the annual symposium. The uses of publication records to study scientific productivity is well-established [19], as are the use of co-authorship as a measure of scientific collaboration [20] and the use of publication rate as a quantification of research progress [21]. Bibliometrics—statistical analyses of publication records—can be potentially used to evaluate individual scientists [22], teams [23], or an entire field of study [24]. It could be expected that any randomly selected group of scientists in the same field of study have co-authored publications. Therefore, we evaluated the incremental impact of the IMGC on the collaboration of its attendees by comparison with other same-size groups of scientists in the same field. Specifically, we used scientific publication records to calculate impact metrics among IMGC attendees compared with null distributions formed by randomly selected same-size subsets of scientists in the same field who were not associated with the IMGC. Finally, to determine the impact of the consortium on the careers of individual scientists, we evaluated their publication histories before and after symposium attendance. In this manuscript, we determine the impact of the IMGC at the levels of both the consortium and the individual scientists.

## Materials and methods

### Data sets

#### IMGC attendee data set

Lists of the full names of attendees at the annual IMGC symposium were obtained for each year from 2004 through 2015 from the consortium’s event manager with the permission of California Dairy Research Foundation, which manages the IMGC. The IMGC Attendee Data Set is available to other researchers through the IMGC Data Access Committee (see Data Availability Statement).

#### Papers published by IMGC attendees

To obtain the papers published by IMGC attendees, the Web of Science Core Collection [25] was queried with the Topic field equal to “milk” OR “lactation” and the Author field populated with the names of the IMGC attendees. In addition, the timespan was set to 2004–2015, and all citation indexes were checked except for the Social Sciences and Arts & Humanities Citation Indexes. This query yielded a total of 7,322 results. An in-house Python script was used to generate text files containing the number of collaborations (defined as the number of times an IMGC attendee appeared on the same paper as another IMGC attendee) and the total number of papers published in the years 2004–2015. The papers included in these files were written by authors who had attended the IMGC symposium in the year of publication or in one of the preceding years.

#### Papers published by all milk scientists

To obtain the papers published by all milk scientists, the same search was done in Web of Science [25], except that the author field was left blank. This search generated *all* the papers on “milk” OR “lactation” published in the timespan 2004–2015. This query yielded 98,649 papers.

### Data analysis

#### Metrics

Using publication records, three metrics of interest were calculated: number of papers published, number of collaborators, and article efficiency. The number of collaborators was determined by co-authorships on publications; each co-author was considered to be a collaborator. The article efficiency is a normalized metric that takes into account the number of citations an article had received as well as its duration in the literature. Given that a paper published many years ago was more likely to be cited than a paper published recently, the normalization is accomplished by dividing the number of citations an article received by the number of years it was available in the literature [26]:

$$article\;efficiency\;=\;\frac{number\;of\;citations}{2016-publication\;year}$$

#### Evaluation of metrics

Metrics were evaluated by permutation testing. A permutation test is a statistical test in which the distribution of the test statistic (e.g. metric) under the null hypothesis is obtained by calculating all possible values of the test statistic when the labels associated with the observed data points are randomized. For both consortia level and individual scientist analyses, p-values were calculated as follows:

$$p-value\;=\;\frac{number\;of\;random\;samples\;with\;a\;larger\;metric\;value\;than\;the\;observed\;value}{total\;number\;of\;random\;samples}$$

#### Evaluation of metrics at consortium level

To determine whether a metric, such as the number of co-authorships, was higher for IMGC attendees than for non-IMGC attendees, the metric was evaluated for both the IMGC attendee population (*n* = 606) and for random permutations of scientists who had published studies on “milk” OR “lactation” in the timespan 2004–2015 but who had not attended the IMGC (*n* = 157,275). For each hypothesis, the null distribution was determined, using in-house Python scripts, based on 20,000 random selections of 606 scientists from among non-IMGC scientists. Note that all IMGC attendees were included in this analysis, even if they had never published a manuscript, whereas the pool of controls (non-IMGC scientists) had published at least one manuscript, biasing the analysis in favor of the null hypothesis.

#### Evaluation of metrics at scientist level

To determine the effect that the IMGC symposium had on individual scientists, an analysis was conducted to compare the three metrics of interest before and after the scientists’ first symposium attendance. For this analysis, the symposium’s effect was quantified through differences in metrics, subtracting the value before first attendance from the value after first attendance:

DifferenceinNumberofPublications=NumberofPublicationsAfterFirstAttendance-NumberofPublicationsBeforeFirstAttendance

DifferenceinNumberofCollaborators=NumberofCollaboratorsAfterFirstAttendance-NumberofCollaboratorsBeforeFirstAttendance

DifferenceinAverageArticleEfficiency=AverageArticleEfficiencyAfterFirstAttendance-AverageArticleEfficiencyBeforeFirstAttendance

Citations and collaborations increase with time. We therefore established timeframes for each scientist to specify the number of years to take into account before and after first conference attendance. These timeframes were established so that the number of years before first attendance equaled the number of years after first attendance, with a maximum timeframe of 5 years before and after (10 years in total). Where the timeframe could not be 5 years, the timeframe was reduced to the maximum possible number of years. For example, the timeframe for first year attendance of 2013 would be reduced to 2 years, since 2015 was the last year taken into account for this analysis. Attendees who had published one or more papers (n = 311) at any time during the years of study (2004–2015) were included in this analysis.

Experiments were repeated with a different set of controls, this time balanced for publication history. After calculating the three differences for the IMGC scientists, the same calculation was done for 20,000 random samples of non-IMGC scientists with similar publication history. Each sample was the same size as the cohort of IMGC scientists. The samples of non-IMGC scientists were created by looping through the IMGC scientists, and randomly selecting a non-IMGC scientist who had the same number of publications as the IMGC scientist in the year of first symposium attendance. Again, IMGC attendees who had not published a paper at any time during the study period (2004–2015) were excluded from the analysis to ensure balanced controls.

#### Software used

Permutation analyses and statistics were conducted using Python code. Distributions of metrics were graphed using R [27], with vertical red lines drawn at the observed value for the IMGC scientists. Networks were visualized and statistics (e.g. diameter) computed using Cytoscape [28].

## Results

### Descriptive analyses

**Fig 1:**
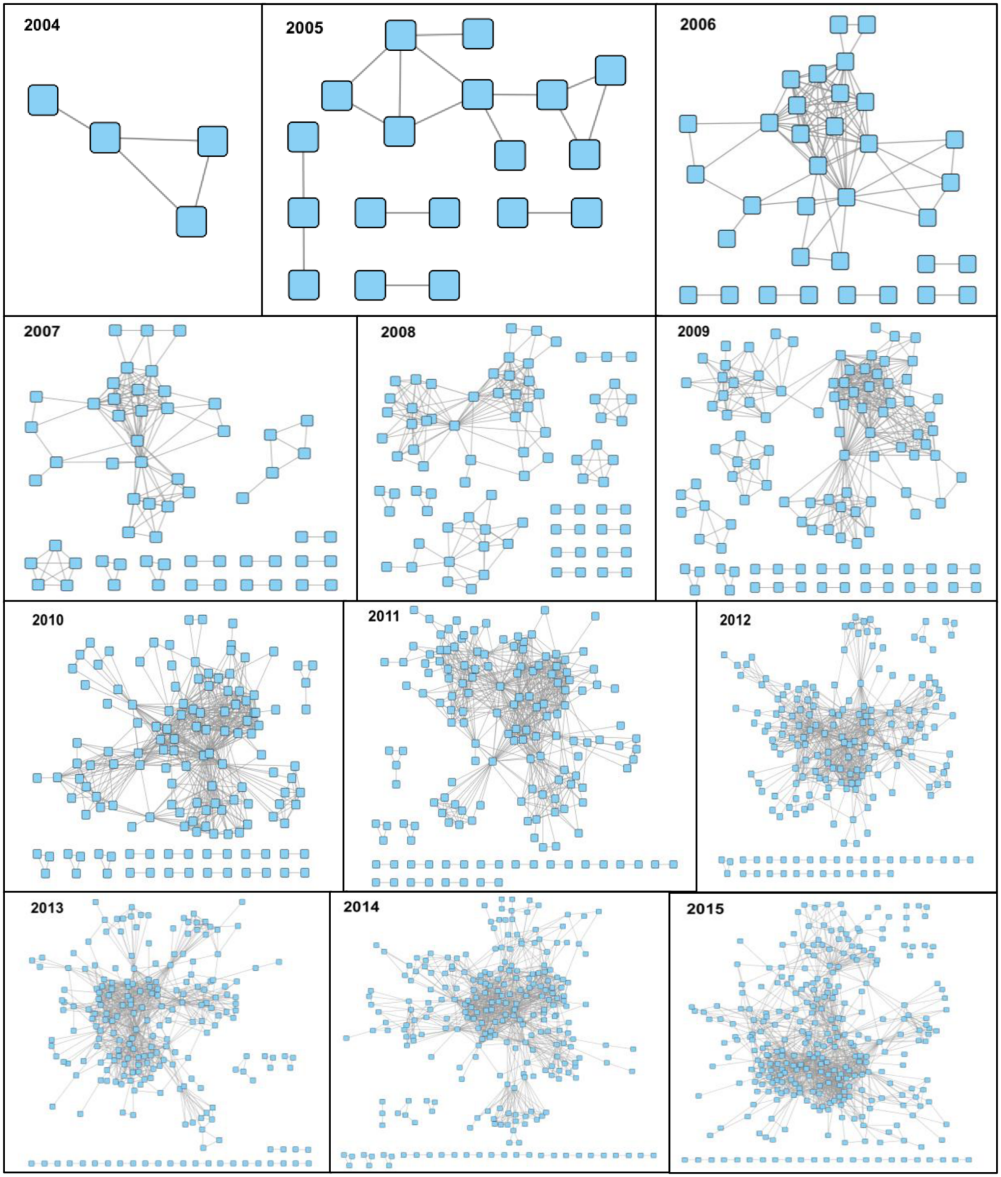
Diagrams of the collaboration networks among IMGC attendees in the years 2004–2015. The collaboration networks were derived from the co-authorships of the attendees’ publications on milk or lactation.

In a social network context, a “node-link” diagram is a type of visualization that captures people as nodes and their relationships as links. A node is represented as a circle or square and the links are represented as lines between the nodes. To determine how the social network of the IMGC changed over the years, attendance records from the annual IMGC symposium were combined with publication records from the Web of Science (see Materials and methods). Each attendee became a node in the network; nodes were linked if two attendees had previously co-authored a publication in milk or lactation science. The networks for the first 12 years of the IMGC annual symposia show that the size of the social networks of IMGC attendees dramatically increased since inception (Fig 1). Between 2005 and 2009, several separate networks formed within the IMGC, largely by geographical distance with each network consisting mainly of scientists from a single country (data not shown). By 2010, co-authorship frequently crossed international boundaries as evidenced by the consortium’s social network turning into a single large network (Fig 1). Since 2010, the consortium’s large single network continued to expand in size with increasing network diameter from 6 to 8. In summary, the size of the IMGC’s social network clearly increased over time.

**Fig 2:**
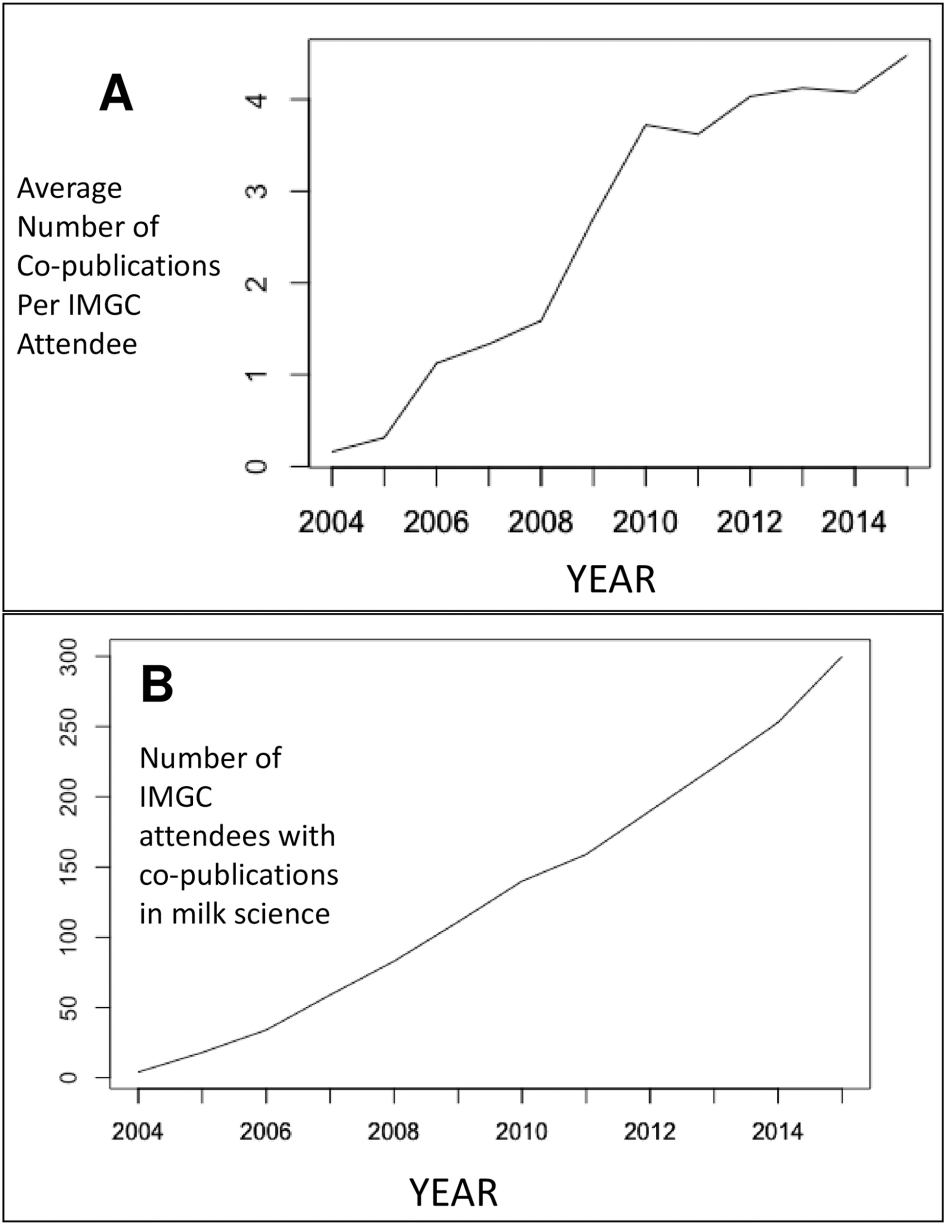
Change in co-publication among IMGC attendees in the years 2004–2015. (A) The average number of co-publications in milk science among IMGC attendees of each annual conference in the years 2004–2015. (B) The cumulative number of IMGC attendees with co-publications in milk science in each year from 2004–2015.

Another way to analyze the same data is to compute the change in co-publication rate among IMGC attendees over time (Fig 2). In 2004, the average IMGC symposium attendee co-published with 0.16 other attendees (Fig 2A). By 2012, the average IMGC symposium attendee co-published with 4 other attendees (Fig 2A). To determine whether the average was being driven by a few highly collaborative attendees, the number of attendees who had co-published with other attendees was computed (Fig 2B). In 2004, only 4 attendees had co-published with other attendees. By 2015, nearly 300 attendees had co-published with other attendees (Fig 2B). This suggests that the IMGC’s social network increased via the collective collaboration of many attendees rather than being driven by very few highly productive people.

### Impact of the IMGC at the consortium level

**Fig 3:**
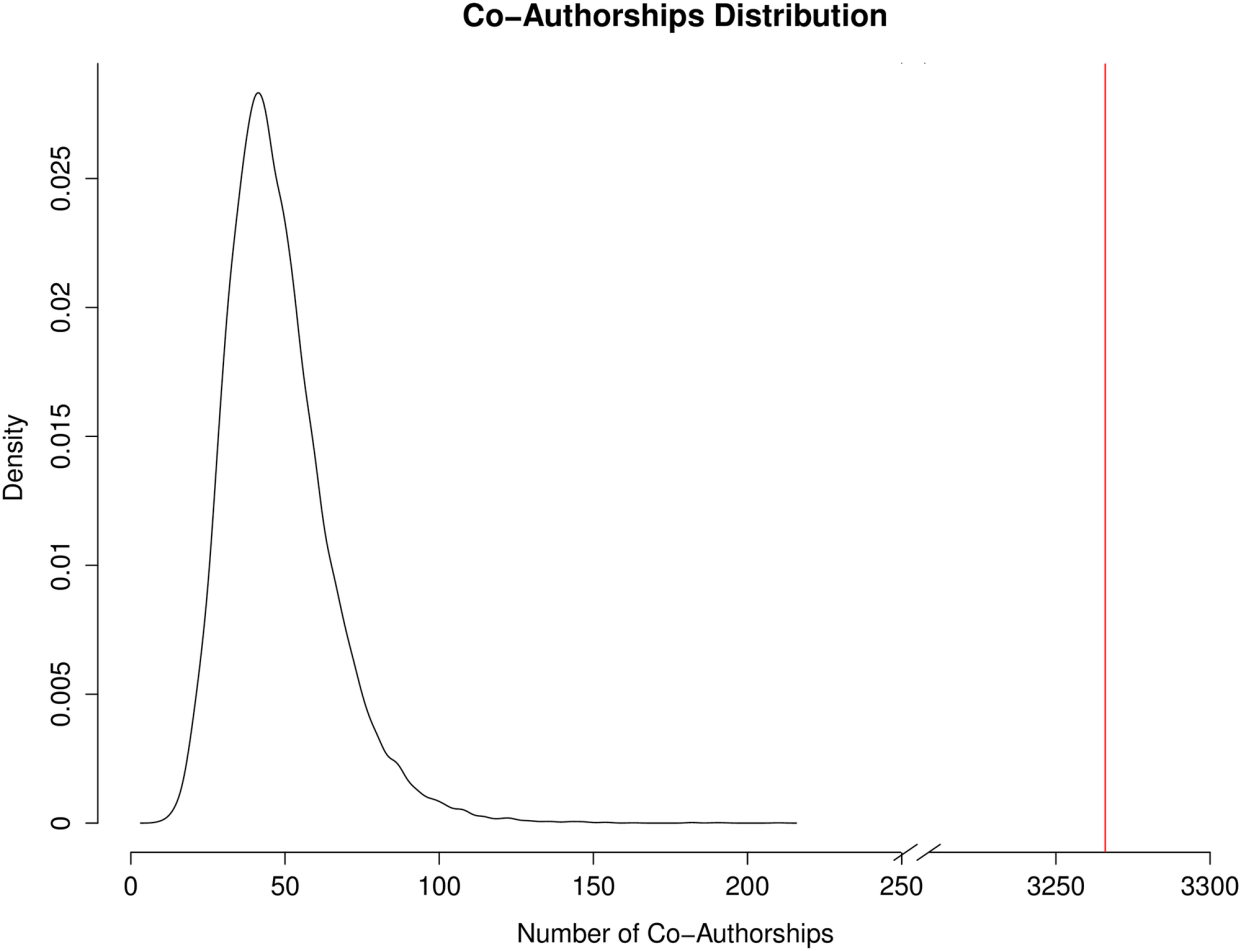
Distribution of number of co-authorships for 20,000 random samples of non-IMGC scientists. The red line is drawn at 3,266, which is the total number of co-authorships among all IMGC attendees (*n* = 606) in the timespan 2004–2015.

**Fig 4:**
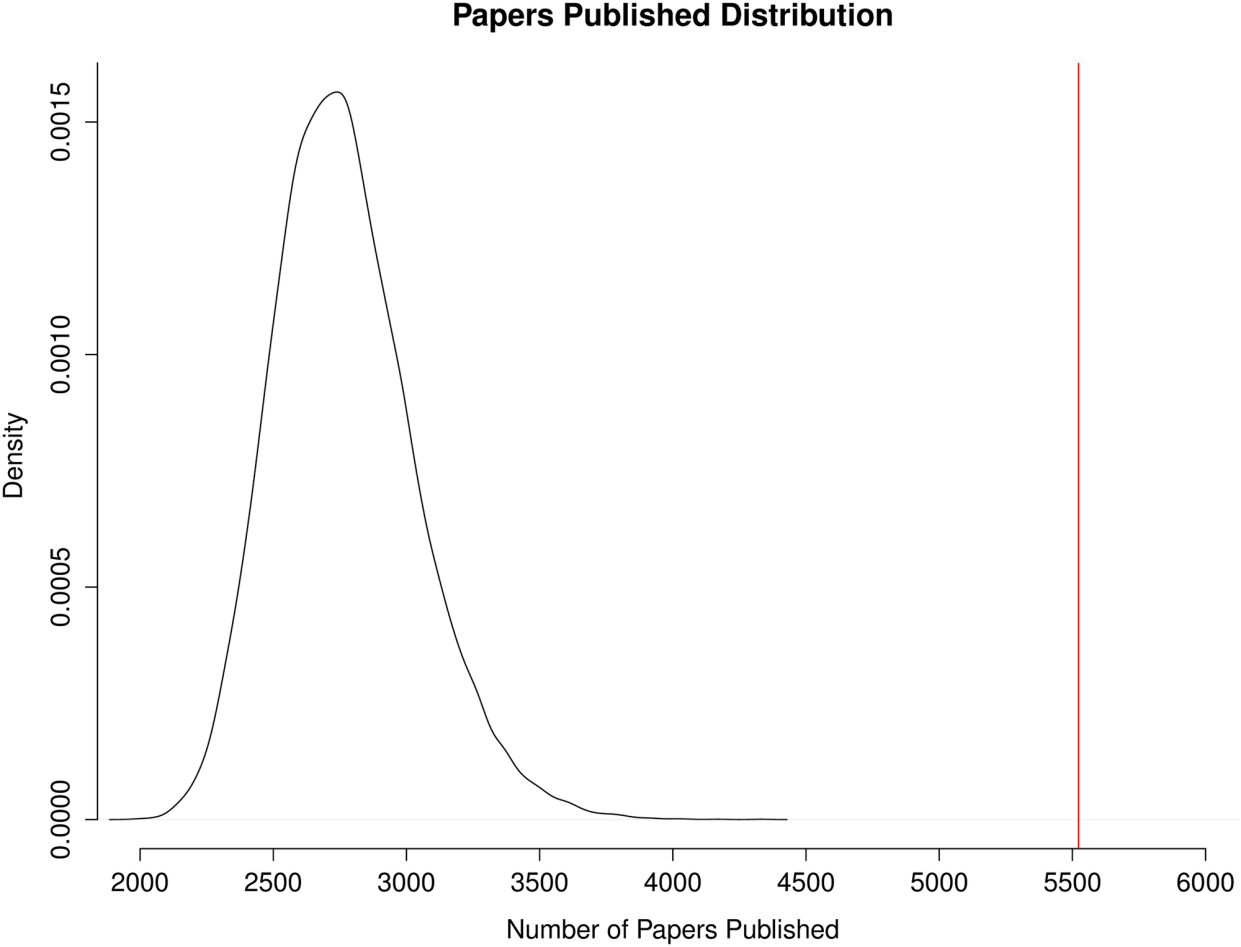
Distribution of number of papers published by 20,000 random samples of non-IMGC scientists. The red line is drawn at 5,523, which is the total number of papers published by all IMGC attendees (*n* = 606) in the timespan 2004–2015.

**Fig 5:**
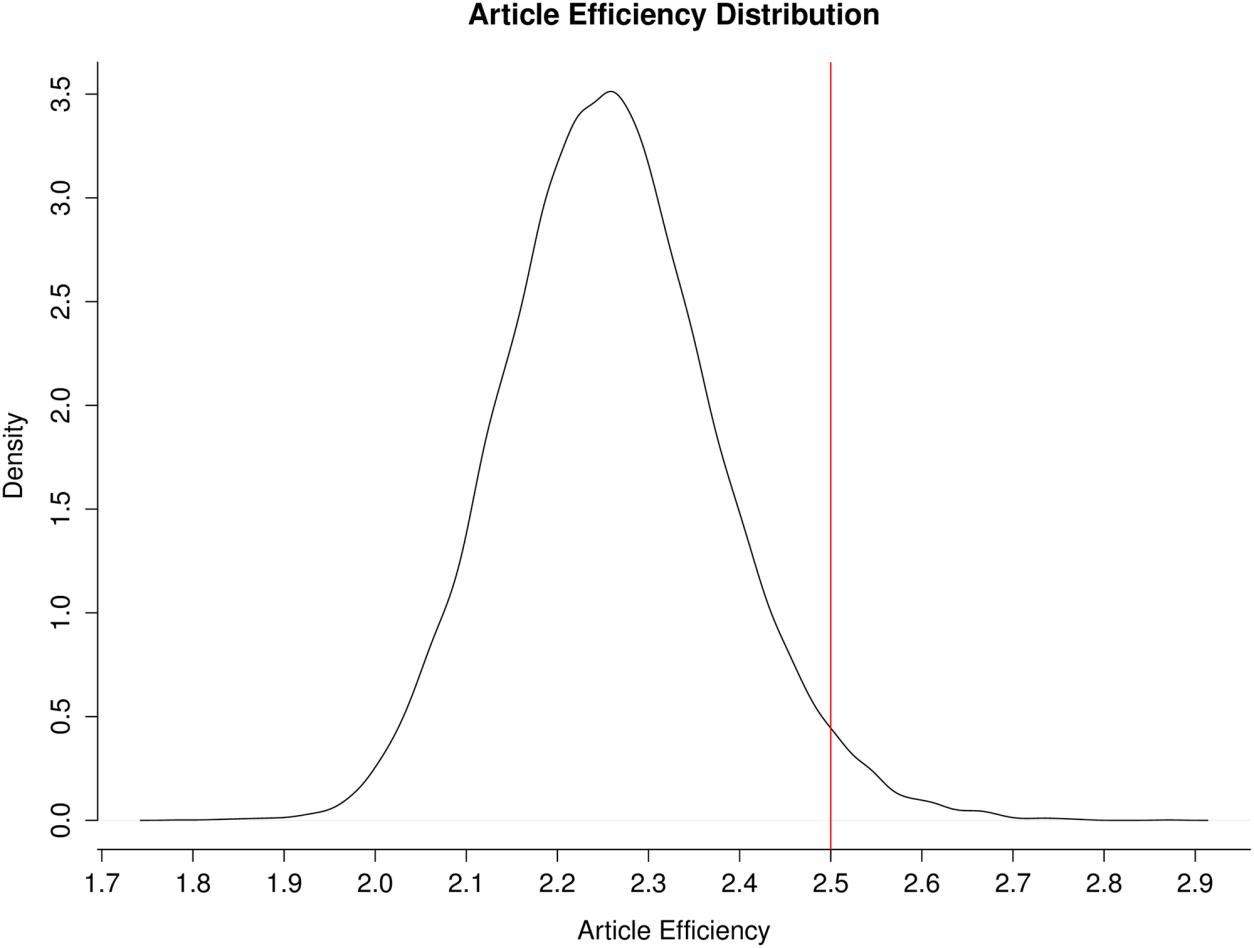
Distribution of article efficiency for 20,000 random samples of non-IMGC scientists. The red line is drawn at 2.50, which is the average efficiency of the articles published by all IMGC attendees (*n* = 606) in the timespan 2004–2015.

To evaluate the impact of the IMGC at the consortium level, analyses were conducted to determine whether or not the IMGC had a significant effect on the amount of collaboration among milk scientists, the productivity of those scientists, and the impact of their papers. The amount of collaboration was measured as a count of co-authorship occurrences in a group of scientists. Scientific productivity was measured as the number of papers published by a group of scientists. The impact of those papers was measured using “article efficiency,” which is a function of both the number of citations the paper has received and how long ago it was published (see Materials and methods). Counts of co-authorships, number of papers published, and article efficiency were each evaluated for the IMGC attendees and random permutations of non-IMGC attendees (see Materials and methods). IMGC attendees had a higher number of co-authorships than expected by chance (*p* = 0) with 3,266 co-authorships compared with a median of just 45 co-authorships among the same number of randomly sampled non-IMGC attendees (Fig 3). In terms of the total count of published papers, IMGC attendees also published more papers than expected by chance (*p* = 0); IMGC attendees published 5,523 papers, compared with an average of 2,770 papers published by the same number of randomly sampled non-IMGC attendees (Fig 4). IMGC attendees had an article efficiency of 2.50, which is higher than the average of 2.26 expected by chance (p = 0.0285, Fig 5). By the measures of the number of published papers, the occurrence of co-authorships on papers, and the impact of those papers, the consortium of IMGC attendees far exceeded what would be expected of a similar number of milk scientists who were not members of the consortium. Thus, the IMGC symposium was associated with increased collaboration, publication output, and article impact.

### Impact of the IMGC at the scientist level

Having found that the IMGC had a positive effect at the level of the consortium, we next investigated whether or not the IMGC symposium has helped its attendees meet new collaborators, increase their productivity in publishing articles (measured by rate of publication), and increase their articles’ impact (measured by article efficiency). In-house Python scripts were written to determine the number of IMGC collaborators each author had before and after their first attendance, the authors’ rates of publication (number of publications per year) before and after their first attendance, and the authors’ article efficiencies before and after their first attendance.

**Fig 6:**
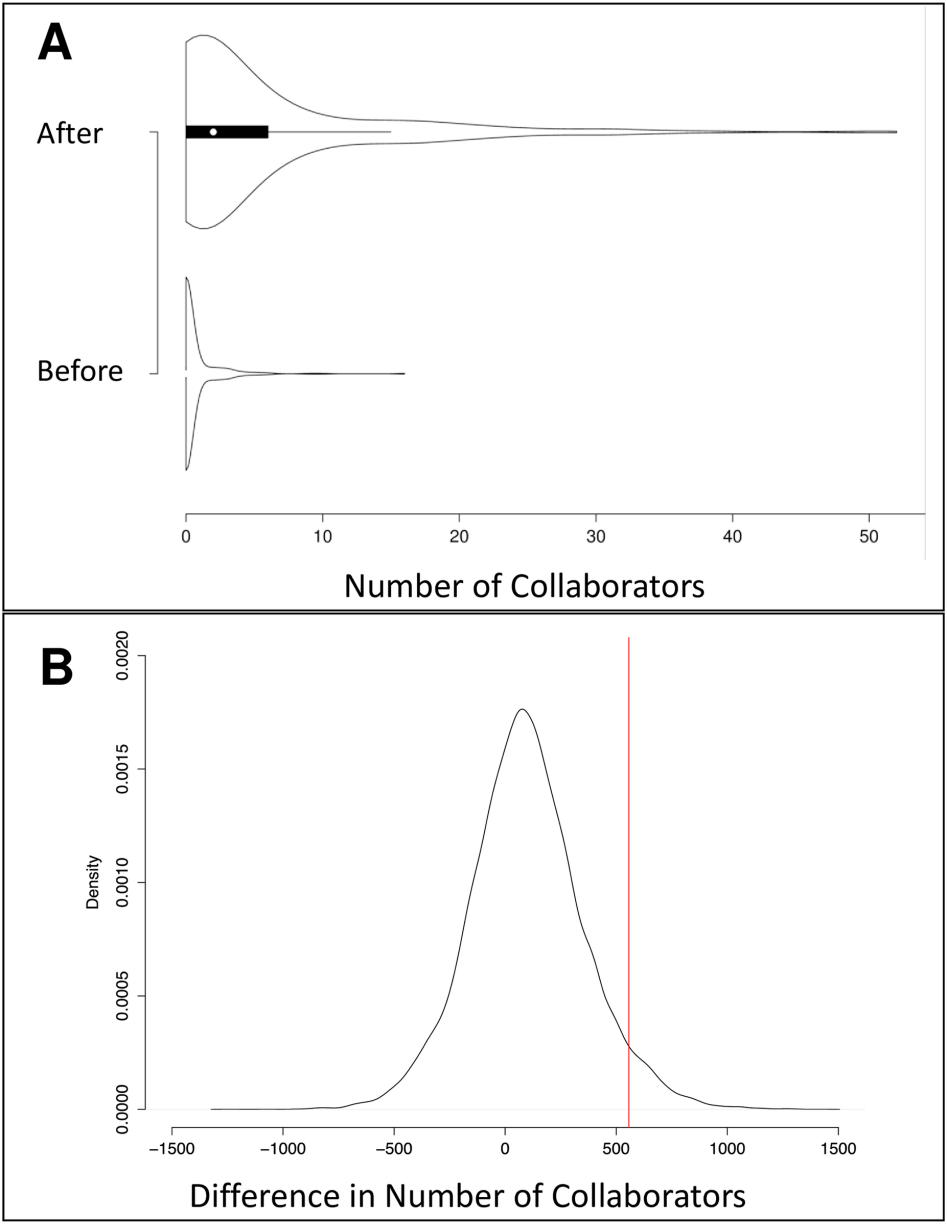
The number of collaborators before and after first IMGC attendance (*n* = 311). (A) Violin plot of number of collaborators before and after first IMGC attendance (*n* = 311). This plot shows that the general distribution of number of collaborators increased after the scientists’ first IMGC attendance. The white circle marks the median, the thick black bar indicates the interquartile range, and the thin black bar indicates the 95% confidence interval. (B) This plot shows the difference in the number of collaborators before and after the first IMGC attendance for all IMGC scientists who had published at least one paper (*n* = 311, red line) compared with the distribution of the same metric for 20,000 random samples of non-IMGC scientist “controls” matched for publication history and years of comparison (black line).

**Fig 7:**
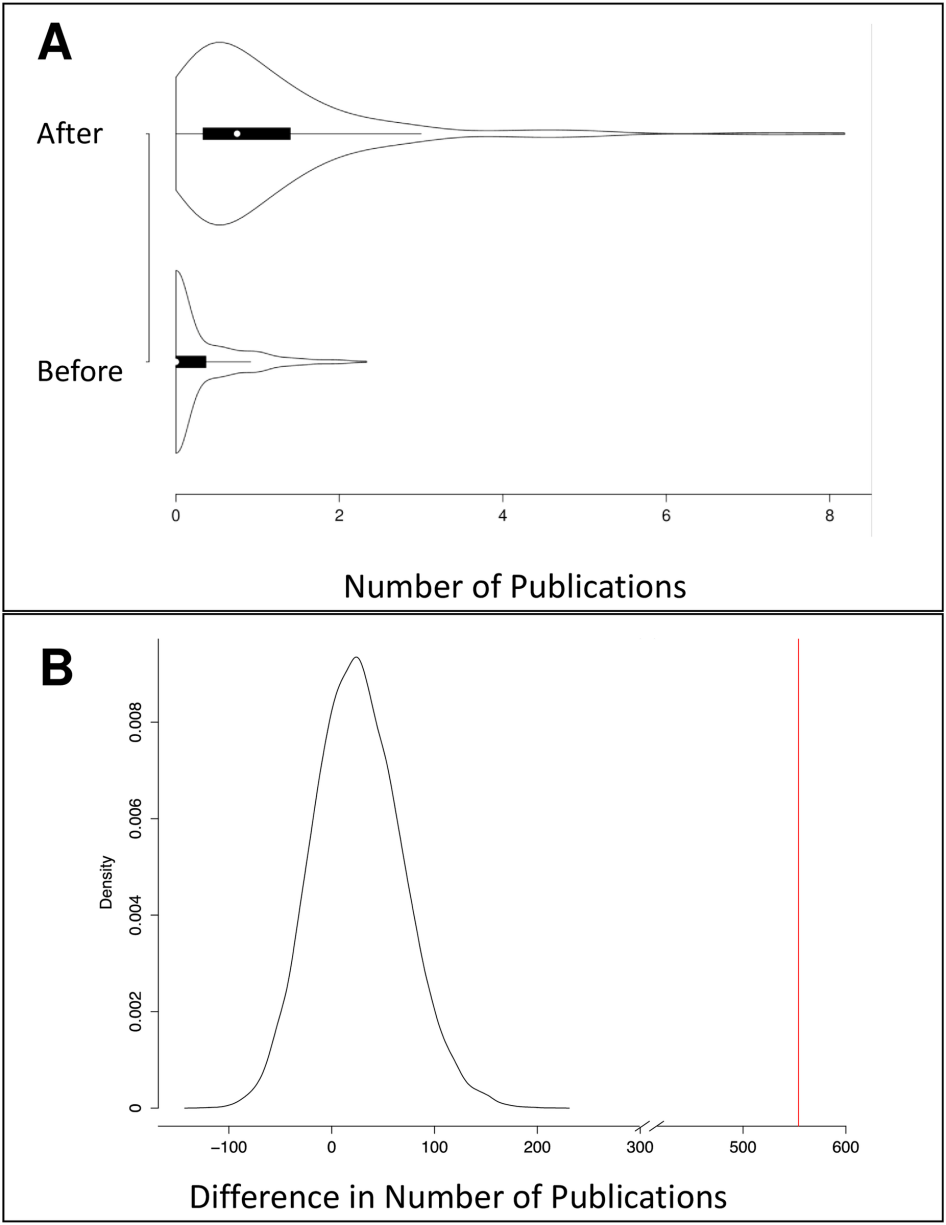
The rate of publication before and after first IMGC attendance (*n* = 311). (A) Violin plot of rate of publication before and after first IMGC attendance (*n* = 311). This plot shows that the general distribution of rate of publication increased after the scientists’ first IMGC attendance. The white circle marks the median, the thick black bar indicates the interquartile range, and the thin black bar indicates the 95% confidence interval. (B) This plot shows the difference in the number of publications before and after the first IMGC attendance for all IMGC scientists who had published at least one paper (*n* = 311, red line) compared with the distribution of the same metric for 20,000 random samples of non-IMGC scientist “controls” matched for publication history and years of comparison (black line).

**Fig 8:**
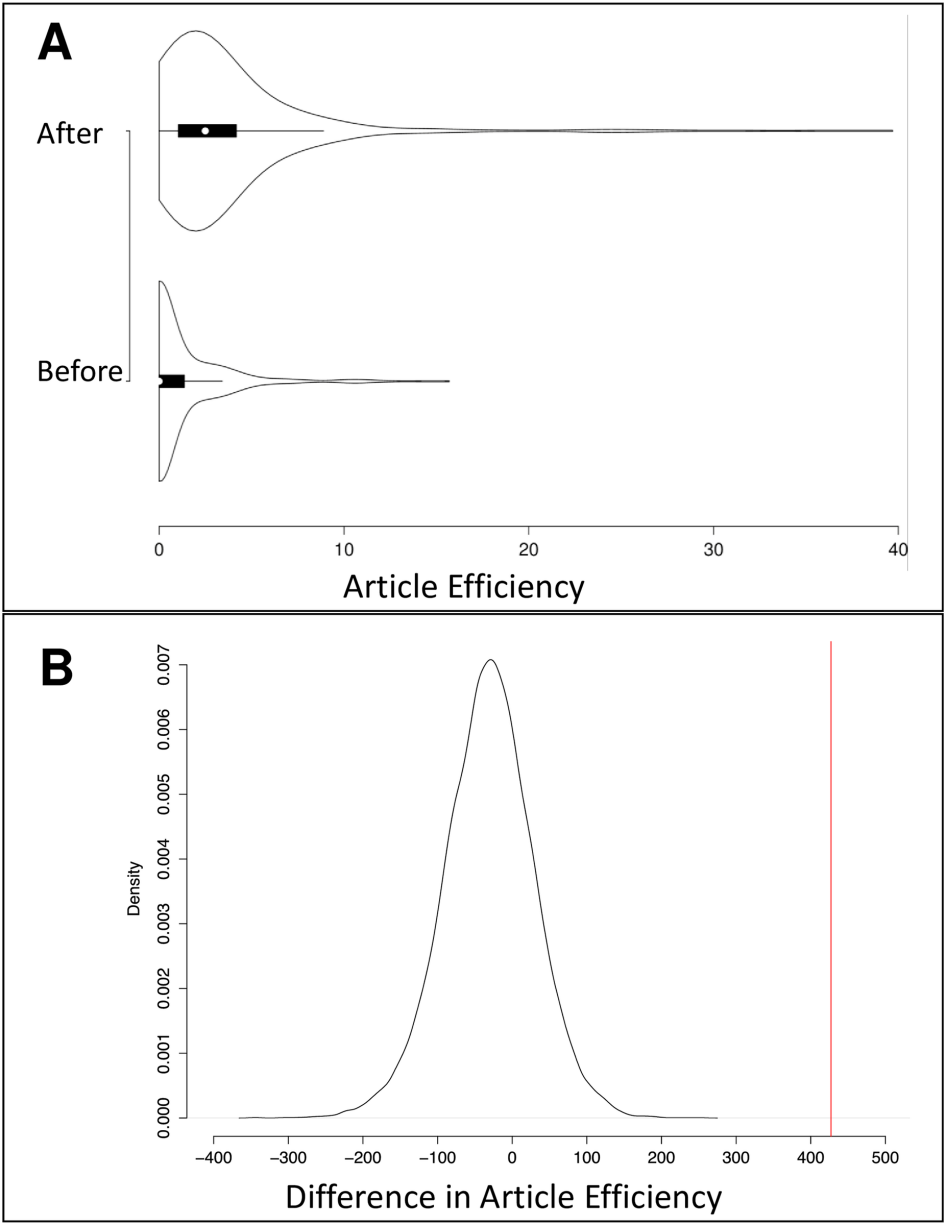
Difference in article efficiency before and after first IMGC attendance (*n* = 311). (A) Violin plot of article efficiency before and after first IMGC attendance (*n* = 311). This plot shows that the general distribution of article efficiency increased after the scientists’ first IMGC attendance. The white circle marks the median, the thick black bar indicates the interquartile range, and the thin black bar indicates the 95% confidence interval. (B) This plot shows the difference in the article efficiency before and after the first IMGC attendance for all IMGC scientists who had published at least one paper (*n* = 311, red line) compared to the distribution of the same metric for 20,000 random samples of non-IMGC scientist “controls” matched for publication history and years of comparison (black line).

A total of 606 people attended the IMGC symposium in the timespan 2004–2015. Of those people, 311 published at least one paper on milk or lactation at some point during the study period of 2004–2015 and were included in scientist-level analyses. For the 311 attendees who published papers on milk or lactation, we evaluated whether or not they had increased their number of collaborators, their rates of publication, and their article efficiencies after attending the conference. The mean, median, and standard deviation of the three measures are shown in Table 1. After attending their first IMGC symposium, 200 of the 311 authors (64.3%) had more collaborators, as measured by co-authorships. Higher publication rates were achieved by 276 of the 311 authors (88.7%) after their first IMGC conference. Of the 311 authors, 231 (74.3%) had higher article efficiencies after attending their first IMGC symposium. On average, these attendees increased their number of collaborators, their rates of publication, and their article efficiencies after attending their first IMGC symposium. Figs 6A, 7A and 8A, respectively, include violin plots that depict the distributions of the number of collaborators, the distributions of the rates of publication, and the distributions of the article efficiencies, respectively. All three plots show that each measure increased on average after the scientists attended their first IMGC symposium.

It is possible that increases in collaborators, productivity, and article impact are merely due to maturity of the scientist that would naturally increase with the passage of time. We therefore compared the observed values of metrics calculated for IMGC attendees with distributions derived from permutation analyses of metrics calculated for non-IMGC scientists with similar publication histories who were in the same field of study. Comparison of the IMGC members with their matched controls suggests that conference attendance did significantly increase collaboration, productivity and article impact (*p* = 0.045, *p* = 0, and *p* = 0, respectively; Figs 6B, 7B and 8B, respectively.). Therefore, we can infer that the IMGC has helped its attendees meet new collaborators, increase their productivity in publishing articles, and increase their articles’ impact.

## Discussion

Since inception, the social network of the IMGC has clearly expanded with smaller groups merging to form larger groups. Milojević [29] posited that the evolution of the sizes of scientific teams happens in two stages. In the first stage, small core teams are formed, likely representing the number of scientists needed to produce a research article. In the second stage, teams expand in size, presumably to conduct research that requires expertise or resources outside of the reach of the core team. Our data supports Milojević’s observations in that the consortium’s social network was initially comprised of small single groups that eventually merged to form a single very large network.

When IMGC symposia attendees were compared with randomly selected scientists in the same field, the impact of the symposia at both the level of the consortium and at the level of the individual scientist was statistically significant for all measures. This suggests that the IMGC has been a successful consortium, despite having a loosely stated mission that is neither directly funded nor mandated in the form of specific publication goals. The mere act of attending an annual scientific meeting in which ongoing research is explained to scientists in other disciplines and to industry is impactful on its own.

The results also suggest that the symposia are beneficial to both the consortium as a whole and to individual attendees. The consortium-level metrics yielded higher co-authorships, higher publication rates, and higher article impact for attendees compared with non-attendees. These metrics—co-authorships, publication rates, and article impact—improved for individual scientists after attendance at their first symposium as well. This observation suggests that the consortium is not merely collecting productive people, but that symposium attendance may also help attendees be more productive.

There are several limitations to the study. First, co-authorship is an imperfect measure of collaboration because some forms of collaboration will not generate co-authored articles [30] whereas there are other collaborations in which very peripheral or indirect forms of interaction between scientists yield co-authored publications [31]. Second, article efficiency may not be the most accurate way of representing article impact. In the economics field, at least half of the citations are received within 5 years of the publication date [32]. If this fact holds true for the field of milk science, then the article efficiency measure would be too low for very old articles. We attempted to ameliorate this problem in the analyses of individual scientists by limiting the timeframe of publications to 5 years before and after the first year of consortium meeting attendance. Third, one must consider that it possible in this type of study that there are unknown confounders and heterogeneity of which we are unaware and therefore not controlled. Using a multivariate analysis of results from survey respondents of the American Political Science Association in 2009, Hesli and Lee [14] identified other factors, such as gender, faculty rank, and PhD program that impacted productivity among political science faculty. Such metadata are not available as part of this data set so the effect of these covariates remains unknown. Fourth, it is impossible to know whether there is truly an effect of the meeting or whether the highly productive scientists are attracted to the meeting. However, this selection bias is somewhat mitigated by matching controls by publication history. Finally, collaboration networks are known to be highly clustered, such that two scientists are much more likely to have collaborated if they have a third common collaborator than are two scientists chosen randomly from the community [33]. Therefore, it is possible that symposia attendees are more likely to co-attend due to pre-existing collaboration than are randomly chosen scientists from the same field. On the other hand, the analyses were biased against the IMGC in that the random selection of individuals only included subjects who have published, whereas the attendee list for the consortium-wide analyses included new scientists and industry members who had never published a paper in the field of study.

Another possible explanation for the relative success of symposia attendees is that they somehow have more research opportunities than other milk scientists due to the nature of their subfield in genetics and/or genomics. To explore the possibility that genome-related research in milk science might have had more research opportunities, we inspected the distribution of milk-related papers published in the “genetics and/or genomics” area vs. other papers among both IMGC attendees and non-attendees (S1 and S2 Files). Surprisingly, there are far more non-genetics/non-genomics papers published by IMGC attendees than in the genetics/genomics area (Figures A and B in S1 File). This same difference between non-genetics/non-genomics papers and genetics/genomics papers is true of non-IMGC attendees (Figures C and D in S1 File). Per scientist, the difference in publication rates between attendees and other milk scientists appear to be similar whether the papers are genetics/genomics (Figures A and C in S2 File) or other papers (Figures B and D in S2 File). In fact, over the time period of study, the difference between the two groups of scientists is more pronounced among non-genetics/non-genomics papers. Therefore, the higher publication rates of the symposia attendees are unlikely to be due to increased research opportunities in their subfield.

An alternate explanation for the relative success of the symposia attendees is that they may be more likely, than other scientists, to have an ongoing research project that produces papers in future years. Many scientific conferences do require that the project being presented has not yet been published and therefore selection of conference attendees from such conferences would introduce a bias. However, the attendees at the IMGC meetings are not required to present pre-publication research. Additionally, attendees are not required to present any project and many do not. For consortium-level analyses, the observed metrics are based on all attendees, regardless of whether or not they are publishing scientists (e.g. some attendees are farmers or non-scientists industry members) whereas the null distribution is based only on publishing scientists, biasing the analysis in favor of the null hypothesis that the meeting has no effect. Despite the fact that the consortium-level analysis includes non-scientist attendees of the IMGC meeting, the meeting still appears to have an effect on scientific productivity.

One aspect of healthy team building is the ability to include both newcomers and incumbents [34]. Each year, as many as 40–50% of the IMGC attendees are newcomers to the symposium. Although we do not know the optimal newcomer rate, it could be surmised, based on the network analysis and publication statistics, that this surprisingly high newcomer ratio is both sustainable and beneficial.

Individuals cannot effectively conduct modern science alone. Scientific meetings enable the sharing of ideas across domains, solving old problems and inspiring creativity. The annual IMGC symposium clearly improved connectivity among scientists in the field, as evidenced by publication records. Previous models of the self-assembly of creative teams have shown that the emergence of such a large connected community marks a phase transition [34]. The IMGC achieved this phase transition within five years of inception and maintained it to the present day, apparently benefiting both the consortium sponsors as a whole as well as attendees.

Our method of utilizing publication records for conducting network analyses could be generalized to study the collaborative effects of other scientific organizations. In particular, publication records can be used to quantify collaboration and productivity among scientists, regardless of the field of study. By harnessing data generated from publication records, demonstrated that the symposia helped bring together milk scientists internationally to form a collaborative scientific community. A barrier to studying scientific consortia more generally has been access to data sets because scientific meeting rosters are not public. In making the IMGC Attendee Data Set available to other researchers, it should be possible in the future to extend the results of our study to determine whether the experiences of this consortium are more generally applicable to other scientific meetings.

## Supporting information

S1 File The number of publications in years 2004–2015 by all IMGC attendees and other milk scientists by area of subfield. The number of publications in years 2004–2015 by (Figures A and B) IMGC attendees and (Figures C and D) Other milk scientists in the areas of (Figure A, Figure C) genetics or genomics or (Figure B, Figure D) not genetics or genomics. Publication counts are from the Web of Science database. Search terms for publications were (Figure A, Figure C) “milk or lactation” AND “genomics or genetics”; (Figure B, Figure D) “milk or lactation” AND NOT “genomics or genetics.”
(PDF)

S2 File The number of publications in years 2004–2015 per each IMGC attendee or other milk scientist by area of subfield. The number of publications in years 2004–2015 per (Figures A and B) IMGC attendee or (Figures C and D) Other milk scientist in the areas of (Figure A, Figure C) genetics or genomics or (Figure B, Figure D) not genetics or genomics. Publication counts are from the Web of Science database. Search terms for publications were (Figure A, Figure C) “milk or lactation” AND “genomics or genetics”; (Figure B, Figure D) “milk or lactation” AND NOT “genomics or genetics.”
(PDF)
