# Supplementary material for: The collaborative effect of scientific meetings: A study of the International Milk Genomics Consortium
Source: PLoS One. 2018 Aug 22;13(8):e0201637. doi: 10.1371/journal.pone.0201637 (PMC6104928; doi:10.1371/journal.pone.0201637)
Supplement: S1 File — The number of publications in years 2004–2015 by (Figures A and B) IMGC attendees and (Figures C and D) Other milk scientists in the areas of (Figure A, Figure C) genetics or genomics or (Figure B, Figure D) not genetics or genomics. Publication counts are from the Web of Science database. Search terms for publications were (Figure A, Figure C) “milk or lactation” AND “genomics or genetics”; (Figure B, Figure D) “milk or lactation” AND NOT “genomics or genetics.” (PDF) [file pone.0201637.s001.pdf]

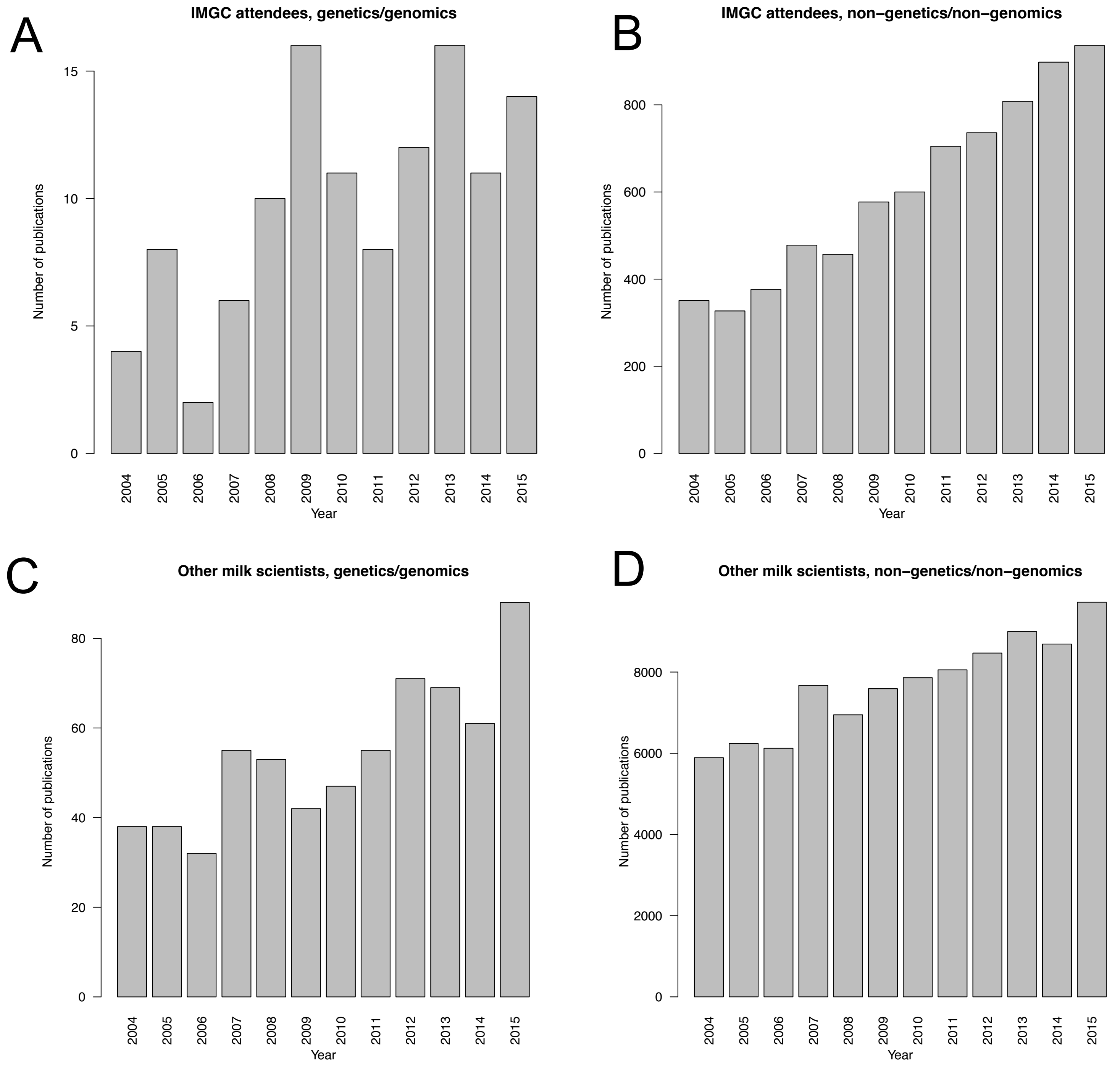
A
IMGC attendees, genetics/genomics
B
IMGC attendees, non–genetics/non–genomics
C
Other milk scientists, genetics/genomics
D
Other milk scientists, non–genetics/non–genomics
Number of publications
Year
2004
2005
2006
2007
2008
2009
2010
2011
2012
2013
2014
2015
0
5
10
15
200
400
600
800
20
40
60
80
2000
4000
6000
8000
