# Supplementary material for: The collaborative effect of scientific meetings: A study of the International Milk Genomics Consortium
Source: PLoS One. 2018 Aug 22;13(8):e0201637. doi: 10.1371/journal.pone.0201637 (PMC6104928; doi:10.1371/journal.pone.0201637)
Supplement: S2 File — The number of publications in years 2004–2015 per (Figures A and B) IMGC attendee or (Figures C and D) Other milk scientist in the areas of (Figure A, Figure C) genetics or genomics or (Figure B, Figure D) not genetics or genomics. Publication counts are from the Web of Science database. Search terms for publications were (Figure A, Figure C) “milk or lactation” AND “genomics or genetics”; (Figure B, Figure D) “milk or lactation” AND NOT “genomics or genetics.” (PDF) [file pone.0201637.s002.pdf]

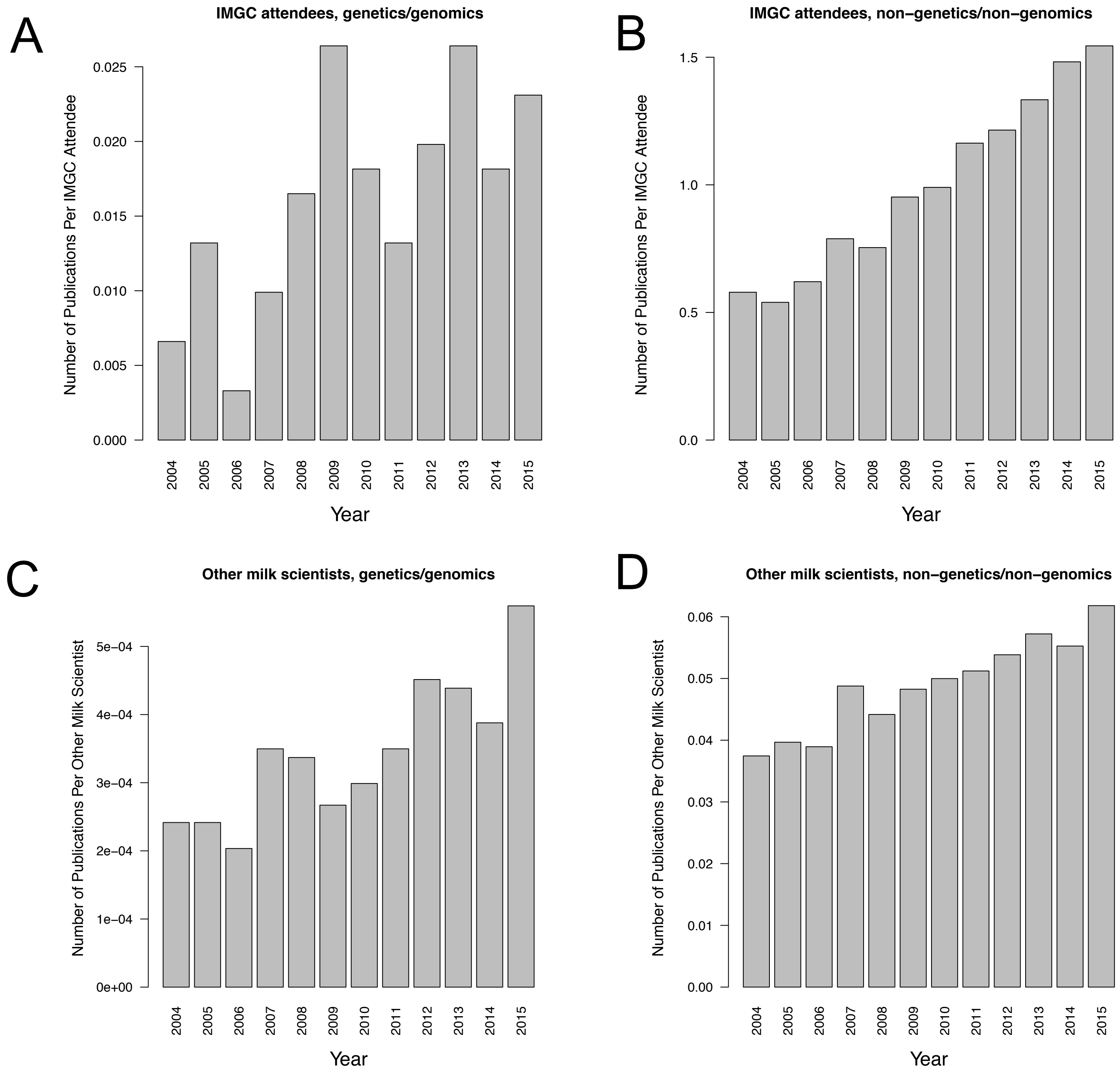

A
IMGC attendees, genetics/genomics
Number of Publications Per IMGC Attendee
0.000
0.005
0.010
0.015
0.020
0.025
2004
2005
2006
2007
2008
2009
2010
2011
2012
2013
2014
2015
Year
B
IMGC attendees, non−genetics/non−genomics
Number of Publications Per IMGC Attendee
0.0
0.5
1.0
1.5
Year
C
Other milk scientists, genetics/genomics
Number of Publications Per Other Milk Scientist
0e+00
1e−04
2e−04
3e−04
4e−04
5e−04
Year
D
Other milk scientists, non−genetics/non−genomics
Number of Publications Per Other Milk Scientist
0.00
0.01
0.02
0.03
0.04
0.05
0.06
Year
